# Donor-derived cytomegalovirus-cytotoxic T lymphocytes and leflunomide successfully control refractory cytomegalovirus infections and disease of multiple sites after allogeneic-hematopoietic stem cell transplantation: A case report

**DOI:** 10.3389/fmed.2022.948210

**Published:** 2022-09-06

**Authors:** Nan Su, Zhenghua Liu, Peng Sun, Feng Gu, Xiaojing Yan, Dali Cai

**Affiliations:** ^1^Department of Hematology, The First Hospital of China Medical University, Shenyang, Liaoning, China; ^2^Department of Ophthalmology, The First Hospital of China Medical University, Shenyang, Liaoning, China

**Keywords:** cytomegalovirus, CTLs, leflunomide, allo-HSCT, case report

## Abstract

Drug-resistant cytomegalovirus (CMV) infection after hematopoietic stem cell transplantation (HSCT) often leads to morbidity and mortality. Several studies have shown that CMV-cytotoxic T lymphocytes (CTLs) can overcome drug-resistant CMV infection, but still many questions remain unanswered. Here, we present a case of refractory CMV infection after allogeneic HSCT (allo-HSCT). Donor-derived CMV-CTLs failed to eliminate the virus in unique peripheral blood on the first application, when 70 mg methylprednisolone (MP) was taken per day. After a second attempt with a combination of 8 mg MP with leflunomide, a complete and persisting clearance of all involved sites, including peripheral blood, urinary system, leptomeninges, and retina, was achieved. To summarize, intravenous infusion of CTLs can eliminate CMV in the oculi and central nervous system (CNS), and a low dosage of 8 mg MP has no interaction with CMV-CTLs.

## Introduction

Cytomegalovirus (CMV) reactivation after hematopoietic stem cell transplantation (HSCT) is a common complication (1). Subsequent therapy with a long-term exposure to anti-CMV drugs might lead to a refractory CMV infection and disease (2). The cause of a refractory infection can be due to either genetic or non-genetic mechanisms. Donor-derived CMV-specific cytotoxic T lymphocytes (CMV-CTLs) facilitate a completely different approach than traditional anti-CMV drugs and are thus a good choice for any mechanism of resistance while supporting a patient to quickly reacquire immunity against CMV, even under immunosuppressive conditions after HSCT (3–5). As a high dose of corticosteroid often contributes to the failure of such therapy (6, 7), a question has arisen about what is the maximal level of corticosteroid, which might have less to no effect on CMV-CTLs? Limited case reports disclose that CMV-CTLs can penetrate blood–brain barrier (BBB) and blood vitreous barrier (8). More clinical studies are required to confirm the effects of CMV-CTLs on CMV infection in the retina and central nervous system (CNS).

In this study, we presented a case with refractory CMV viremia, concurrent infections in both the urinary and CNS, and additional CMV retinitis after nearly 3-month application of foscarnet and anti-CMV immunoglobulin for CMV viremia. After two infusions of donor-derived CMV-CTLs and systemic administration of leflunomide, the patient reached a complete viral clearance in all involved organs and tissues, including the retina and CNS. The second CMV-CTLs infusion under the administration of 8 mg methylprednisolone (MP) per day proved the most effective. We proposed the application of CMV-CTLs as a treatment for resistant CMV disease even in the retina and CNS, better under the condition of a low dose of corticosteroid.

## Case presentation

We presented a case of 29-year-old man, diagnosed with acute myeloid leukemia and myelodysplasia-related changes (AML-MRCs) transformed from myelodysplastic syndrome (MDS). The patient (CMV-IgM-negative and CMV-IgG-positive) without hematological remission due to chemo-resistance underwent related 7/10 human leukocyte antigen (HLA) matched peripheral blood HSCT from his mother (CMV-IgM-negative and CMV-IgG-positive) after conditioning with modified BuCy2 in August 2020. In the process of HSCT, the patient was routinely immunosuppressed with rabbit anti-human thymoglobulin (ATG), cyclosporine (CSA), methotrexate (MTX), and mortimecofenate (MMF) for graft-versus-host disease (GVHD) prophylaxis (9). On day + 16 and day + 20 after HSCT, a neutrophil and platelet engraftment was successful, respectively, as well as hematological and immunophenotypic complete remission. However, on day + 26 after transplantation, he developed skin acute graft-versus-host disease (aGVHD) grade II, for which we prescribed him 2 mg/kg MP.

**FIGURE 1:**
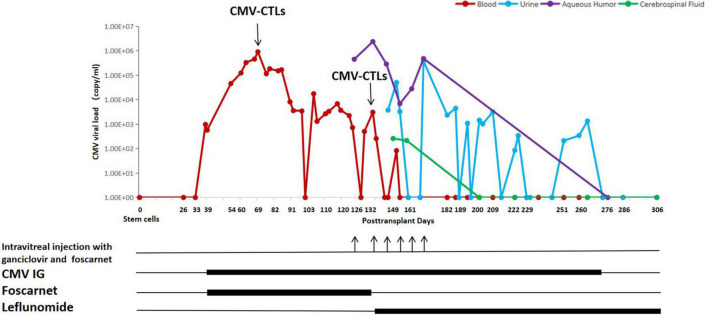
CMV viral load and antiviral treatments of the patient.

On day + 39, a CMV viremia with 9.62×10^2^ DNA copies/ml got detected by PCR on a whole blood sample, which triggered a pre-emptive anti-CMV therapy. The CMV viral load and the treatment timeline are shown in Figure 1. Due to pancytopenia, he received foscarnet 60 mg/kg q8h and CMV immunoglobulin 100 mg/kg qod. Subsequently, on day + 40, the aGVHD was well controlled, consequently reducing MP by 10% weekly.

A resistance to foscarnet was suspected on day + 69, as the CMV DNAemia titer increased up to 8.94 × 10^5^ copies/ml. Therefore, the first dose of 7.23 × 10^9^ donor-derived CMV-CTLs [produced by Beijing ICELL Biotechnology Co., a detailed procedure of generation of CMV-CTLs was previously described (10)] was intravenously infused, while the dosage of MP was 70 mg/day. Three weeks after infusion of CMV-CTLs, the CMV titer decreased significantly to 1.0^∼^6.0 × 10^3^ copies/ml but failed to further drop, even though continuously treated with foscarnet and CMV immunoglobulin. To ascertain the reason behind the observed resistance, a CMV genotyping of blood sample was performed on day + 120 where a UL54 T961S mutation was identified, an up to now undefined mutation to drug resistance. Due to the patient’s complaint of blurry vision in both eyes, he underwent a fundus examination on day + 126, which revealed hemorrhage and frost-like changes in vessels. The CMV titer in the aqueous humor of the right eye was 4.46 × 10^5^ copies/ml, but other viruses were negative, including herpes simplex virus, Epstein-Barr virus, varicella zoster virus, and other human herpes viruses. Therefore, a CMV-retinitis (CMVR) was diagnosed. Clinically poor response to standard therapy and occurrence of CMV retinitis indicated a condition refractory to foscarnet. Hence, the patient received weekly intravitreal injection with 2 mg/0.05 ml ganciclovir in addition to 0.6 mg/0.05 ml foscarnet, as well as systemic anti-CMV therapies. The latter included leflunomide, a medication against rheumatoid arthritis, chosen as a treatment option for resistant CMV infection together with the second infusion of CMV-CTLs. Leflunomide was taken orally at 100 mg/day for 3 days, hereafter reduced to 20 mg/day continuously. On day + 132, the patient received the second dose 5.00 × 10^9^ of donor-derived CMV-CTLs; meanwhile, MP was taken at the dosage of 8 mg/day. Twelve days after the second infusion of CMV-CTLs, CMV copy numbers in the blood finally decreased, indicating CMV-CTLs and leflunomide went into effect. However, copy numbers of CMV DNA in aqueous humor remained unchanged; additionally, the eyesight did not improve, which led to the refusal of further intravitreal injections by the patient. Longer follow-up showed a continuous decrease of CMV DNA in the blood implying sufficient expansion of CMV CTLs and quick reconstitution of immunity against the virus. Meanwhile, a steady decline in retina inflammation indicated an improvement in retinitis.

On day + 149, the patient was unable to urinate spontaneously without urine catheterization. Ultrasound and CT examination of the urinary system as well as the neuromagnetic resonance examination showed no abnormalities. Urine and cerebrospinal fluid were also tested for viruses and leukemia. The results showed positive CMV DNA in both body fluids, very high levels of protein, and no leukemia cells in cerebrospinal fluid. Hence, a CMV infection in the urinary system and leptomeninges were diagnosed. Since previous case reports demonstrated that CMV-CTLs can potentially completely eliminate CMV in the CNS, and valid reconstitution of antiviral immunity in the blood of our patient was confirmed, we continued with the leflunomide, CMV immunoglobulin, and supportive care. The clinical symptoms gradually abated and disappeared within a month. In addition, the CMV titer turned negative in cerebrospinal fluid on day + 200 (68 days after the second CTLs) and in urine on day + 273 (141 days after the second CTLs). In view of the significant clinical improvements, the patient agreed to an intravitreal puncture again on day + 276 (144 days after the second CTLs) to re-examine CMV DNA in aqueous humor, which was negative.

Henceforth, the patient is in continuous hematological remission, and his blood, urine, cerebrospinal fluid, and aqueous fluid have all been continuously negative for CMV.

## Discussion

Our patient showed severe refractory CMV infections under the treatment with a high dose of immunosuppression due to aGVHD after allo-HSCT. Initially, an increase of more than one log fold change of virus DNA copies in blood and a subsequent occurrence of CMVR under long-term therapy with foscarnet both indicated the resistance to the drug, even though no known defined mutation on UL54 and UL97 was found in the blood sample. Finally, additional resistant evidence showed that ganciclovir and foscarnet failed to decrease the copies of CMV in aqueous humor after weekly bilateral intraocular injections. The difficult question remained to choose a therapy in order to effectively treat the patient. As it was not feasible to run sequencing on a sample of aqueous humor, we could not select sensitive anti-CMV drugs, according to UL54 and UL97 gene mutations. In addition, neither systemic administration of ganciclovir nor maribavir and letermovir could be applied, due to a poor neutrophil level or unavailability.

This is why we concluded CMV-CTLs would be the best option for our patient, as the mechanism of this kind of immune cells is completely different from those of traditional anti-CMV drugs (11). They attack CMV-infected cells to stop CMV amplification through direct cytolysis and indirect cell death, by releasing perforin/granzyme. Until recently, several studies have verified that donor-derived CMV-CTLs can overcome drug resistance of CMV after HSCT (2, 4, 5). In addition, limited case reports disclose that CMV-CTLs in the blood can efficiently eliminate CMV inside the oculus and CNS, even though separated by BBB and blood-ocular barrier (8, 12). Besides the perfect efficacy of the immune cells, donor-derived CMV-CTLs are safe for transplant recipients without the induction or aggravation of GVHD (13). Donor-derived CMV-CTLs can be quickly manufactured *in vitro* if the donor is ever infected by CMV. Fortunately, lymphocytes from the patient’s mother were appropriate for producing CMV-CTLs. We immediately tried the first dose of CMV-CTLs in our patient, when refractory status was suspected, which resulted in partial response in the blood, confirming CTLs’ effectiveness. These promising results lead us to the decision, which we also try CMV-CTLs again, when later a refractory CMVR was diagnosed.

In addition to CMV-CTLs, we simultaneously applied oral leflunomide. It can penetrate the BBB and inhibit virion assembly of CMV, overcoming drug resistance via a completely different mode as ganciclovir and foscarnet (14). Research discloses that leflunomide could be measured in aqueous humor as the patient takes it orally. The level could be as high as 4.1 μg/ml (15). Clinically, two retrospective studies have verified that leflunomide, in combination with or without other medicines, has a potential against resistant CMV infection, including CMV retinitis (14, 15). As a final result, the combination of CMV-CTLs and leflunomide permanently eliminated CMV in all infected organs and tissues in our patient.

It is well known that post-transplant patients exposed to corticosteroids are highly susceptible to CMV infection (16, 17). A high dose of corticosteroid definitely has a negative influence by inhibiting the proliferation of donor-derived CMV-CTLs *in vivo* (6, 7). Then, what is the maximal level of corticosteroid which almost has no interaction with CTLs? In our case, the first infusion led to an only partial reduction of CMV load under 1.5 mg/kg MP and later progressed with refractory retinitis as well as urinary and cerebral infections. However, after the second infusion, when MP was decreased to 8 mg per day, we observed a persistent negative CMV measurement in blood, as well as a complete cure of all infections of involved sites until this writing, which indicates the continuous expansion of CTLs in blood and 8 mg MP per day has no influence on CMV-CTLs.

In conclusion, our case suggests that donor-derived CMV-CTLs, combined with leflunomide, can effectively and safely control the refractory CMV infection at multiple sites and can lead to persistent remission. Quick recovery of CMV-specific T cells by infusion of donor-derived CMV-CTLs dominantly controls infection and disease in the retina and CNS. Doses equal to or lower than 8 mg per day of MP do not prevent the proliferation of CTLs.

## Data availability statement

The original contributions presented in this study are included in the article/supplementary material, further inquiries can be directed to the corresponding author.

## Ethics statement

Ethical review and approval was not required for the study on human participants in accordance with the local legislation and institutional requirements. Written informed consent was obtained from the individual for the publication of any potentially identifiable images or data included in this article.

## Author contributions

NS and DC guided the treatment of this case and analyzed the patient’s data regarding the hematological disease and the transplant. NS was a major contributor in writing the manuscript. NS and ZL reviewed all related literature. XY and DC critically revised the manuscript. PS and FG performed fundus examination, aqueous humor puncture, and intravitreal injection. All authors read and approved the final manuscript.
